# A retrospective cohort study examining the outcomes of patients who present for fertility care and exceed a set body mass index threshold for treatment

**DOI:** 10.1016/j.xfre.2024.08.010

**Published:** 2024-09-07

**Authors:** Olutunmike Kuyoro, Michal Mia Shalamov, Cailey Brogan, Randi Goldman

**Affiliations:** a Northwell, New Hyde Park, New York; b Department of Obstetrics and Gynecology, Northwell Health Fertility, New York, New York; c Zucker School of Medicine, Uniondale, New York; d American Medical Program, Tel Aviv University, Tel Aviv, Israel

**Keywords:** Obesity, ART, healthcare barriers, weight loss

## Abstract

**Objective:**

To evaluate the characteristics of patients who exceeded the body mass index (BMI) threshold for fertility treatment at their initial visit and identify those for whom treatment would be constrained.

**Design:**

Retrospective cohort study.

**Setting:**

Academic medical center.

**Patient(s):**

All new patients who presented for infertility treatment at an academic center between January 2020 and December 2022 and had BMI measured and recorded.

**Main Outcome Measure(s):**

Likelihood of weight loss and treatment initiation for patients who exceed a set BMI threshold of 40 kg/m^2^.

**Result(s):**

Of the 1,268 patients who had their BMI recorded at initial visit, 48% identified as non-Hispanic White, 15% as non-Hispanic Black, 13% as Asian, 11% as Hispanic, 0.2% as Native American, 4% as other; 9% were of unknown race/ethnicity. Overall, 6% of women exceeded the 40 kg/m^2^ cutoff. Among Latino women, 7.5% exceeded the cutoff; among non-Hispanic Black women, 12% exceeded the cutoff. These percentages were greater than the percentage of non-Hispanic White women who exceeded the BMI cutoff (4.8%).

**Conclusion(s):**

Body mass index thresholds disproportionately affect the ability of ethnic minorities to use fertility treatment and could potentially be worsening barriers to care these population of patients already face.

In the United States, prepregnancy obesity impacts approximately one-third of reproductive-aged women (1) and has been shown to be associated with rates of infertility three times higher than non-obese women (2). Time to pregnancy is additionally increased in overweight and obese women when compared with their normal-weight counterparts (3). The relationship between obesity and infertility is complex and multifactorial. Mechanisms implicated include disruptions in the hypothalamic-pituitary-ovarian axis resulting in anovulation (4), aberrant oocyte recruitment and quality (5), impaired embryo quality, and diminished endometrial receptivity (6). Women within the class II and III obesity groups (body mass index [BMI], 35 kg/m^2^ to ≥40 kg/m^2^) are most likely to seek assistance to achieve pregnancy, yet are least likely to receive and use fertility care services in the United States (7). This likely reflects national and global policies that have advocated for the restriction of fertility services to women below a certain BMI threshold (7, 8, 9).

The highest rates of elevated BMI are found among underrepresented minorities (10), and it is evident that, as a single parameter, BMI is a relatively poor predictor of health and tends to estimate health risks for ethnic minorities less accurately (11, 12). When obesity is defined based solely on BMI, there is substantial misclassification of individuals into weight classifications, such that ethnic minorities are more likely to be misclassified. For example, Burkhauser et al. (12) showed that not only are African Americans more likely to be wrongly classified by BMI, but the disparity in obesity rates between African American and White women is cut in half when percent body fat is instead used to define obesity (13).

Although there is no universal BMI cutoff above which all Society for Assisted Reproductive Technology-fertility clinics deny in vitro fertilization (IVF) treatment, approximately 65% of programs in the United States have a self-imposed cutoff that typically ranges between 35 and 45 kg/m^2^ (7). As rates of obesity are higher in Latino and non-Hispanic Black patients compared with non-Hispanic White patients, BMI thresholds for fertility care may therefore inadvertently discriminate against populations of patients who already face barriers to access to care (14).

Given that restrictions on fertility treatment by BMI have the potential to amplify disparities in access to care, the objective of this study was to evaluate the characteristics of patients who exceeded the BMI threshold for fertility treatment at their initial visit and identify those for whom treatment would be constrained outright. This study additionally sought to determine the likelihood of required weight loss before treatment initiation and to elucidate associated demographic factors that may underpin differences in weight management.

## Materials and methods

This was a retrospective cohort study of new patients with infertility presenting to an academic fertility center in New York between January 2020 and December 2022. This academic center is comprised of five sites with two IVF laboratories. All patients have their height and weight measured at the time of their initial in-person visit, and their BMI is automatically documented in the electronic medical record. During this study’s timeframe, the practice had implemented a BMI cutoff for IVF treatment of 40 kg/m^2^. Patients who desired treatment with IVF and had a BMI of >40 kg/m^2^ were advised to lose weight before treatment initiation, often in conjunction with local Centers for Weight Management, and were allowed to proceed with IVF once their BMI was 40 kg/m^2^ or less. Although there was no strict BMI cutoff for patients seeking treatment with intrauterine insemination (IUI), patients with BMIs in the obese range were typically counseled regarding weight loss before treatment initiation and deferral of IUI treatment for the purpose of weight loss was patient and provider-dependent.

Sociodemographic characteristics were collected from an electronic medical record including self-reported race and ethnicity, age, and medical comorbidities. Patients’ BMI at the time of initial presentation, any changes in BMI over clinical course, and details regarding whether the patient initiated any treatment course within a year of presentation were also collected and recorded. Using ANOVA, we analyzed whether there were significant differences in ages between the different racial groups and used Tukey post-hoc tests to determine the specific groups within which the potential differences may exist. In addition from this data, the likelihood of having a BMI >40 kg/m^2^ at the new patient visit was compared according to race. Logistic regression models were also developed from the collected data and used to determine the association between race and likelihood of weight loss; and likelihood of treatment initiation (IUI and IVF), with *P<.*05 defining significance. Given that many programs in the United States and globally employ a cutoff of ≥35 kg/m^2^, we secondarily examined the effect that a putative cutoff of 35 kg/m^2^ would have on restricting treatment access in our patient population.

Statistical analysis of the odds ratio (OR) involved the use of the Wald statistic, which was derived from the regression slope coefficient and its standard error. The 95% confidence interval (CI) was calculated by applying the approximate normal distribution to the estimates of the logistic regression parameters. This study was deemed exempt by the Northwell Health/Hofstra School of Medicine Institutional Review Board.

## Results

Between January 2020 to December 2022, a total of 2,103 women attended a new patient visit, of which 1,268 (60%) had an in-person visit at which their BMI was recorded and were thus included in this study. Among the entire cohort, 48% identified as non-Hispanic White, 15% as non-Hispanic Black, 13% as Asian, 11% as Latino, 0.2% as Native American, 4% as other; 9% were of unknown race and ethnicity (Table 1).

**Table 1:** Baseline characteristics of women presenting for initial evaluation.

Mean Age/BMI	Total	Asian	Latino	Non-Hispanic Black	Non-Hispanic White	Native American	Other	Unknown
Count (% of total)	1,268	162 (13%)	134 (11%)	191 (15%)	610 (48%)	3 (0.2%)	50 (4%)	117 (9%)
Age (y)								
Mean (SD)	35.5	35.01 (4.71)	36.22 (4.99)	37.14 (4.92)	34.93 (4.60)	35.67 (6.11)	34.18 (5.17)	35.38 (5.46)
BMI (kg/m^2^)	27.9	26.07	28.74	31.01	27.13	28.64	28.50	27.85

*Note:* BMI = body mass index.

The average age at the time of new patient visit was 35.5 years and varied according to race, as determined by one-way ANOVA, *F*(6, 1261) = 6.389, *P<.*001. Of patients with known race, Black patients had the highest age at time of presentation with a mean of 37 years. Post-hoc testing revealed significant differences in ages between Black and White patients as well as between Black and Asian patients (*P*≤.001), but not between any of the other races.

Among the cohort, 75 patients (6%) were found to have a BMI of ≥40 kg/m^2^. Of these patients, 29 (39%) were non-Hispanic White, which represented 5% of the total White population (Table 2). Although there was a similar number of non-Hispanic Black patients with BMI >40 kg/m^2^ (N = 24), this represented a higher proportion of the total non-Hispanic Black patient population at 12% – approximately double that of the non-Hispanic White population of patients. Ten Latino patients had a BMI ≥40 kg/m^2^, which represented 7.5% of the total Hispanic population, and there were five Asian patients with BMIs ≥40 kg/m^2^, which represented 3% of the total Asian population with recorded BMIs.

**Table 2:** Ethnic distribution of patients with body mass index >40 kg/m^2^.

Counts	Total	Asian	Hispanic	Non-Hispanic Black	Non-Hispanic White	Native American	Other	Unknown
% of total	75	5 (6.7%)	10 (13.2%)	24 (32%)	29 (38.7%)	0	2 (2.7%)	5 (6.7%)
% of total number of women within the respective ethnic group	—	3%	7.5%	12%	4.8%	0	4%	4.3%

Among patients with a BMI ≥40 kg/m^2^, 24% initiated fertility treatment within a year of initial presentation. The majority of these were of non-Hispanic White race (N = 12), representing 41% of the non-Hispanic White population with BMI ≥40 kg/m^2^ (N = 29). A smaller percentage of non-Hispanic Black (11%) and Latino (11%) patients with a starting BMI of ≥40 kg/m^2^ initiated fertility treatment within a year of presentation, respectively representing 8% and 20% of the total non-Hispanic Black (N = 24) and Latino (N = 10) populations with BMI ≥40 kg/m^2^.

**Figure 1:**
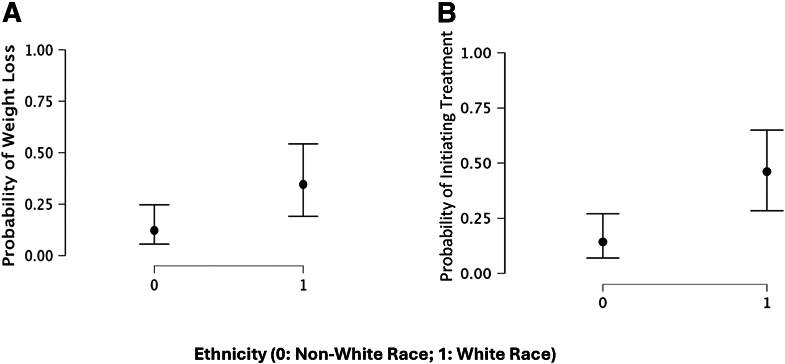
(**A**) Likelihood of weight loss. (**B**) Likelihood of initiating treatment.

Fewer than one in five patients (N = 14, 19%) who had a starting BMI of ≥40 kg/m^2^ lost any amount of weight within 1 year of initial presentation. Of these patients, 9 (64%) were non-Hispanic White, which represented 31% of the non-Hispanic White population with BMI ≥40 kg/m^2^. Using logistic regression, the likelihood of losing any amount of weight and initiating treatment based on race were examined (Fig. 1A, B). Patients were more likely to lose any amount of weight if they were non-Hispanic White (OR, 3.79; 95% CI, –2.8 to –1.1; *P=.*026) compared with other races. Patients were more likely to initiate treatment if they were non-Hispanic White (OR, 5.14; 95% CI, –2.5 to –0.9; *P*=.004) vs. any other race. The likelihood of exceeding the 40 kg/m^2^ cutoff based on ethnicity was also examined. Although not statistically significant, patients were more likely to exceed the 40 kg/m^2^ threshold if they were not White (OR, 1.55; *P=.*071).

Using a more restrictive BMI cutoff of ≥35 kg/m^2^, the number of patients who would not have met the criteria for IVF treatment nearly tripled from 75 (6%) to 206 (16%). When stratified by ethnic group, the Hispanic (17%) and non-Hispanic Black (29.5%) ethnic groups still had a higher proportion of their total respective populations fall within this BMI group than the non-Hispanic White patients (14.6%) (Table 3). The likelihood of patients exceeding a BMI cutoff of 35 kg/m^2^ was greater if they were not White (OR, 1.36; 95% CI, 0.001–0.620; *P=.*049).

**Table 3:** Ethnic distribution of patients with body mass index ≥35 kg/m^2^.

Counts	Total	Asian	Latino	Non-Hispanic Black	Non-Hispanic White	Native American	Other	Unknown
% of total	206	13 (6.3%)	23 (11%)	57 (28%)	89 (43%)	0	11 (5.3%)	13 (6.4%)
% of total number of women within the respective ethnic group	—	8%	17%	29.5%	14.6%	0	22%	11%

## Discussion

There is a preponderance of evidence that depicts the reduced rates at which underrepresented minorities seek and access fertility care when compared with non-Hispanic White patients (14, 15, 16, 17). In this retrospective cohort study of new patients who presented for fertility care and exceeded an established BMI cutoff, our findings demonstrate that BMI cutoffs may further exacerbate these disparities in access to care. The rationing of fertility treatment based on BMI has the potential to deny care to a significant portion of patients who may otherwise be eligible for care, disproportionately affecting these underrepresented populations.

Consistent with previous findings, our study showed that ethnic minorities tend to be older than their non-Hispanic White equivalents at time of presentation. This finding has been attributed to a variety of factors, one of which is delay in specialist referral, which may be due to conscious or unconscious physician bias regarding who should or should not become a parent, and thus who warrants fertility treatment (14). In our analyses, we show that that these barriers to access may be further compounded by strict BMI cutoffs because although a higher proportion of the ethnic minorities seeking care exceeded BMI thresholds both at a more stringent cutoff of 35 kg/m^2^ and a higher one of 40 kg/m^2^, these patients were also less likely to lose any weight and therefore less likely to initiate any treatment after initial presentation than their non-Hispanic White counterparts. This finding is in line with a study by DeLany et al. (18) which showed that despite similar adherence to weight loss interventions, non-Hispanic Black women achieved less body weight loss than non-Hispanic women, which may be related to intrinsically different metabolic processes and energy requirements between the two groups.

Per National Institute of Health guidelines on obesity (19), a constant caloric deficit of approximately 3,500 kcal/wk can be expected to yield a weight loss of approximately 0.5 kg/wk. Applying this guideline to the Latino patients who exceeded the BMI cutoff in this study, with an average BMI of 44.6 and an average age of 36.7 years, it would take one such patient a minimum of at least a year to meet the <40 kg/m^2^ threshold, at which point she would be close to 38 years of age with subsequent diminished oocyte quantity and quality (9). The negative effects of this decrease in oocyte quality and quantity on live birth rates are unlikely to be mitigated by weight loss (20).

This projection, taken together with the findings of a recent randomized controlled trial that showed weight loss before treatment did not ostensibly improve live birth rates in women undergoing assisted reproductive technology treatment (21), suggests that the requirement of weight loss before permitting access to treatment may be of dubious benefit.

Advocates for BMI cutoffs often cite concerns regarding safe administration of anesthesia during oocyte retrievals to this patient population; however, a recent study showed that in the appropriate clinical setting, retrievals may be safely performed in patients with class III and IV obesity (22). The American Society for Reproductive Medicine, therefore, currently advocates for a holistic outlook on BMI (23), one that balances the ability of individual programs to safely provide assisted reproductive technology treatment although being mindful of the ethical principles of medicine as they pertain to the patient. For this reason, at our center where retrievals are performed in an outpatient setting, in consultation with our anesthesia team, we have raised our BMI threshold for IVF to 45kg/m^2^. Although we are optimistic that this may ameliorate some of the biases and barriers engendered by an otherwise lower threshold, we acknowledge that it likely does not fully eliminate them.

A strength of our study was that although patients were from a single academic site, this site is comprised of multiple centers distributed throughout several New York boroughs, allowing for some generalizability of findings. A limitation of this study was the relatively small number of women in the cohort of patients who exceeded the 40 kg/m^2^ threshold, limiting more robust statistical analyses. Although data for this study was collected over a 2-year period, weight loss often requires a longer length of time and as such, follow-up data collected over more time may allow for more robust analyses. Additionally, it is possible that there were other reasons that patients did not initiate treatment or pursue IVF unrelated to weight/BMI, including personal preference, cost, and other sociodemographic variables that could have acted as barriers to care. Nonetheless, to our knowledge, this is the first study detailing the impact that BMI cutoffs may have on access to fertility treatment, according to race and ethnicity.

Given the findings of this study, which demonstrate that there is an overrepresentation of obese patients who exceed weight thresholds among ethnic minorities presenting for fertility care, and consequently decreasing their likelihood of accessing fertility treatment, it is vital that clinicians are mindful of weight management practice patterns that may lead to further underutilization of fertility services.

## CRediT Authorship Contribution Statement

**Olutunmike Kuyoro:** Conceptualization, Data curation, Formal analysis, Methodology, Writing - original draft, Writing - review & editing. **Michal Mia Shalamov:** Data curation. **Cailey Brogan:** Data curation. **Randi Goldman:** Conceptualization, Formal analysis, Supervision, Writing - review & editing.

## Declaration of Interests

O.K. has nothing to disclose. M.S. has nothing to disclose. C.B. has nothing to disclose. R.G. is a peer reviewer for UpToDate.
